# Whole-exome sequencing for a more accurate diagnosis of intraductal papillary neoplasms of the bile duct

**DOI:** 10.1093/gastro/goab014

**Published:** 2021-04-10

**Authors:** Tomoaki Matsumori, Norimitsu Uza, Nobuyuki Kakiuchi, Toshihiro Morita, Yoshihiro Nishikawa, Masahiro Shiokawa, Kojiro Taura, Yuzo Kodama, Hiroshi Seno

**Affiliations:** 1 Department of Gastroenterology and Hepatology, Graduate School of Medicine, Kyoto University, Kyoto, Japan; 2 Department of Phathology and Tumor Biology, Kyoto University, Kyoto, Japan; 3 Department of Hepato-Biliary-Pancreatic Surgery, Graduate school of Medicine, Kyoto University, Kyoto, Japan; 4 Department of Gastroenterology, Kobe University, Kobe, Japan

## Introduction

According to the World Health Organization (WHO, 2018), intraductal papillary neoplasms of the bile duct (IPNB) are characterized as polypoid masses in dilated bile ducts; additionally, invasive IPNB occasionally presents with a nodular surface or mass formation [1, 2]. The IPNB diagnostic criteria are, however, often ambiguous, e.g. whether IPNB is considered a type of cancer and whether it must have mucus production are debatable [3]. Therefore, establishing new IPNB diagnostic criteria based on an alternative approach, such as whole-exome sequencing, is required. Here, we present a case of IPNB with atypical features on which we performed a detailed genetic analysis to determine the genetic, morphological, and histological features of IPNB in this case.

## Case presentation

**Figure 1.**
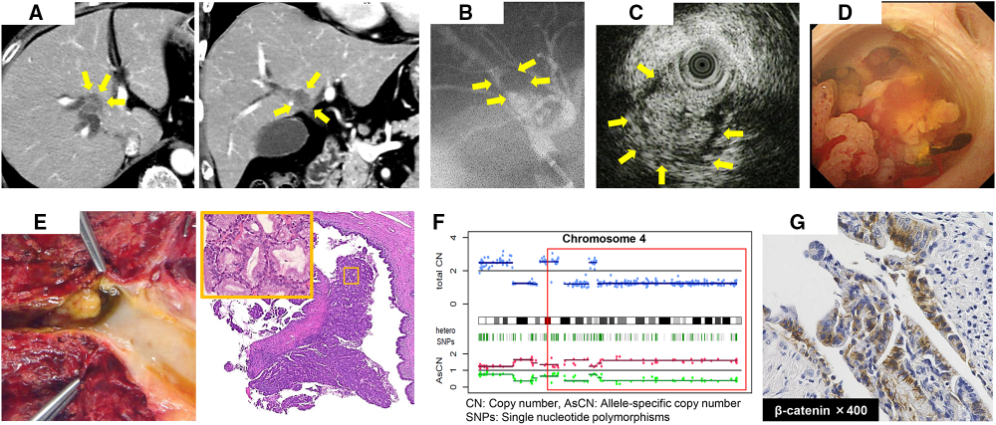
Imaging and immunohistological findings of the intraductal papillary neoplasms of the bile-duct case. (A) Contrast-enhanced computed tomography imaging features. Yellow arrows point to the tumor lesion. (B) Endoscopic retrograde cholangiography imaging features. Yellow arrows indicate the defect area caused by the tumor. (C) Intraductal ultrasonography imaging features. Yellow arrows highlight the tumor lesion. (D) Per-oral cholangioscopy imaging features. (E) Macroscopic image and hematoxylin and eosin staining features. (F) Copy-number alterations in chromosome 4. The red box shows the long arm of human chromosome 4. (G) Immunohistochemistry staining of β-catenin in the tumor tissues.

A 68-year-old woman with dilatation of the right hepatic bile duct was referred to our hospital. Contrast-enhanced computed tomography images revealed a tumor in the right hepatic bile duct (**Figure 1A**). Endoscopic retrograde cholangiography further showed a defect with a smooth surface localized in the dilated right hepatic bile duct (**Figure 1B**). Additionally, intraductal ultrasonography revealed that the tumor mass filled the dilated right hepatic bile duct and that the tumor surface showed a multinodular pattern (**Figure 1C**). Based on the fluoroscopic biopsy results, we suspected IPNB. Per-oral cholangioscopy (POCS) was also performed and confirmed that the tumor had a multinodular surface and moved lambently in response to water injection (**Figure 1D**). A POCS-guided biopsy allowed a definitive diagnosis of IPNB; horizontal spreading was not detected, and a right hepatectomy with extrahepatic bile-duct resection was performed. Histopathological examination of the resected specimen confirmed IPNB with a multinodular surface, mainly consisting of pyloric-gland-like cells with low-grade atypia (**Figure 1E**). The results of immunohistochemistry staining further revealed that the cells expressed MUC6, but not MUC1, MUC2, or MUC5AC; the Ki-67 labeling index was <1%. Additionally, whole-exome sequencing, in the context of tumor cells extracted from the surgically resected specimen, revealed that *CTNNB1* and *NFE2L2* mutations were the significant oncogenic mutations (**Table 1**).

**Table 1.** Somatic mutations revealed by whole-exome sequencing in this case-study

G**ene**	Chr	Start	Ref	Alternate	Mutation type	Amino-acid change	VAF
LRIT3	4	110791232	C	T	Nonsense	NM_198506: p.Q443X	0.646
MBNL1	3	152150623	A	G	Missense	NM_001314057: p.M98V	0.46
RNF31	14	24621187	C	G	Missense	NM_017999p.L706V	0.435
PER1	17	8044602	G	C	Synonymous	NM_002616: p.L1219L	0.418
ATP1A1	1	116936319	–	G	Frameshift insertion	NM_001160234: p.L514fs	0.417
NFE2L2	2	178098815	T	C	Missense	NM_006164: p.D77G	0.416
WRN	8	30958470	T	A	Missense	NM_000553: p.M696K	0.416
GREB1	2	11780550	G	A	Synonymous	NM_014668: p.P1940P	0.41
PKHD1	6	51798942	G	A	Synonymous	NM_138694: p.F2029F	0.4
ZNF582	19	56896509	G	C	Missense	NM_144690: p.H93D	0.397
DBF4	7	87525791	C	T	Synonymous	NM_006716: p.G200G	0.391
CDHR3	7	105662725	A	G	Missense	NM_152750: p.Y636C	0.382
AVL9	7	32609638	C	G	Missense	NM_015060: p.L408V	0.366
WNK1	12	988975	T	A	Synonymous	NM_018979: p.T870T	0.364
SETD2	3	47125677	T	A	Missense	NM_014159: p.S1865C	0.363
DCAKD	17	43112197	C	T	Synonymous	NM_024819: p.Q19Q	0.356
CTNNB1	3	41266134	C	G	Missense	NM_001904: p.P44R	0.344
ZNF544	19	58772421	TGTT	–	Frameshift deletion	NM_014480: p.L150fs	0.324
TRIM16	17	15532476	T	C	Missense	NM_006470: p.H383R	0.31
SPRED1	15	38614538	ACGTTTCA	–	Frameshift deletion	NM_152594: p.T102fs	0.289
CPA4	7	129944403	C	T	Missense	NM_016352: p.P157L	0.287
ZEB1	10	31810108	T	C	Synonymous	NM_030751: p.A615A	0.283
MBD1	18	47806362	T	C	Missense	NM_002384: p.M1V	0.273
AASS	7	121738917	T	C	Synonymous	NM_005763: p.E470E	0.236
RNF34	12	121858078	GAT	–	Inframe deletion	NM_194271: p.224_224del	0.235
FNDC3B	3	172003752	T	–	frameshift deletion	NM_022763: p.L276fs	0.212
FAT3	11	92533227	C	T	Nonsense	NM_001008781: p.R2350X	0.205
ZNRF3	22	29445928	C	T	Missense	NM_032173: p.R487C	0.119
MACF1	1	39818806	G	T	Missense	NM_012090: p.G1714V	0.102
LRP1B	2	142237983	C	T	Missense	NM_018557: p.E109K	0.089
PJA2	5	108691675	C	A	Missense	NM_014819: p.V569L	0.076
EZH1	17	40871163	T	C	Missense	NM_001991: p.M243V	0.073
SPTAN1	9	131395509	–	C	Frameshift insertion	NM_003127: p.D2439fs	0.051

Genes in red: hot-spot mutations in well-known oncogenes. Chr, chromosome; Ref., reference; VAF, variant allele frequency.

*LRIT3* (located on the long arm of human chromosome 4) showed two distinct gene alterations: a nonsense mutation and a loss of heterozygosity (LOH) (**Table 1** and **Figure 1F**). Moreover, most of the other mutated genes listed in the table were passenger mutations with no functional amino-acid changes. Currently, the patient is alive without tumor recurrence 5 years after the surgery.

## Discussion

In this case, the tumor filled the hepatic bile duct and caused dilatation of the peripheral bile duct. Though the tumor nearly met the WHO diagnostic criteria for IPNB, some features were atypical. Despite the absence of an invasive lesion, the surface properties exhibited a nodular pattern. Moreover, the cells constituting the tumor were primarily pyloric-gland-like cells. Furthermore, in the biliary system, pyloric-gland adenomas are often found in the context of gallbladder polyps, but of IPNB [4]. Although some whole-exome sequencing-based studies on bile-duct cancers have been reported, and *KRAS* and *TP53* are listed as important gene variants, most analyses were performed in mixed patients with IPNB and bile-duct cancers [5]. Aoki *et al.* recently performed targeted capture sequencing of cancer-driver genes in the context of IPNB [3]; however IPNB-specific gene mutations could not be identified via target gene analysis alone. In the present case, whole-exome sequencing of the tumor revealed somatic mutations in well-known cancer-driver genes such as *CTNNB1* and *NFE2L2*; interestingly, mutations frequently identified in bile-duct cancers and IPNB, such as *KRAS* and *GNAS* mutations, were not found (**Table 1**). Therefore, collectively, this IPNB case was both phenotypically and genotypically atypical, suggesting that the identified genetic mutations were potentially involved in the formation of the characteristic morphological and histological features. Furthermore, these results indicate that IPNB is heterogeneous and the current diagnostic criteria are inadequate.

*CTNNB1* is an important constituent gene in the Wnt/β-catenin pathway. Although the Wnt pathway is known to be activated in bile-duct cancers, *CTNNB1* mutation is not commonly reported in patients with cholangiocarcinoma and IPNB [3, 6]. Moreover, genetic mutation in *CTNNB1* is involved in the formation of pyloric-gland polyps in the stomach and gall bladder [7]. Of note, immunohistochemistry analysis in the context of the case we report here revealed that β-catenin was expressed in both the cytoplasm and the nuclei of IPNB cells (**Figure 1G**). In hepatocellular carcinoma, membrane-bound β-catenin is observed in precancerous lesions or low-grade cancers, whereas β-catenin translocates into the nucleus as cancer progresses [8]. Considering the histologically low-grade atypia and the distribution of β-catenin observed in our case, IPNB may progress from a benign to malignant phenotype through β-catenin nuclear translocation.

*NFE2L2*, also known as *NRF2*, is one of the major genes involved in the cellular antioxidant response, regulating the basal and inducible expression of cell-proliferation- and carcinogenesis-related genes, including *NOTCH1*, *NPNT*, *VMPR1A*, *IFG1*, and *JAG1* [9]. The mutations in *CTNNB1* and *NFE2L2* identified in the case we have reported here are listed as oncogenic mutations in the ***Catalogue of Somatic Mutations in Cancer*** (https://cancer.sanger.ac.uk/cosmic). Curiously, although *LRIT3* has not been reported as a cancer driver, it showed the highest mutant allele frequency in our case with two-hit mutations, a nonsense mutation, and an LOH; therefore, we cannot exclude the notion that such mutations were involved in the formation of IPNB. In fact, such a hypothesis is supported by the fact that *LRIT3* is a regulator of *FGFR1* [10], one of the driver genes of cholangiocarcinoma. Although other mutations may have played driver roles, we could not find any reports supporting their association with the phenotype observed in this case. Therefore, large-scale genetic analysis of IPNB is further needed to completely understand the molecular pathology in IPNB and to establish clear diagnostic criteria.

## Conclusions

We identified three distinct genetic alternations that may be involved in the formation of IPNB with an atypical phenotype. The accumulation of similar cases and large-scale analyses is, however, still necessary to validate the role of these genes in the pathogenesis of IPNB and to establish clear IPNB diagnostic/classification criteria.

## Authors’ Contributions

T.M., N.U., N.K., T.M., Y.N., M.S., K.T., and Y.K. performed the clinical examinations and experiments, and analysed the data. T.M. wrote the manuscript, and N.U., Y.K., and H.S. revised it.

## Funding

None.
